# Co-occurrence of myositis and neuropathy after anti-CD30 therapy in a late-adolescent Hodgkin lymphoma patient

**DOI:** 10.1186/s40478-025-02056-2

**Published:** 2025-06-28

**Authors:** Adela Della Marina, Lydia Rink, Andreas Hentschel, Michael M. Schündeln, Christopher Nelke, Heike Kölbel, Calvin Tucht, Vera Dobelmann, Tobias Ruck, Tim Hagenacker, Teresinha Evangelista, Ulrike Schara-Schmidt, Andreas Roos

**Affiliations:** 1 Department of Pediatric Neurology, Center for Neuromuscular Disorders in Children and Adolescents, University Hospital Essen, University of Duisburg-Essen, Essen, Germany; 2 Division of Pediatric Hematology and Oncology, Department of Pediatrics III, University Hospital Essen, University of Duisburg-Essen, Essen, Germany; 3 Leibniz-Institut für Analytische Wissenschaften -ISAS- E.V., Dortmund, Germany; 4 Department of Neurology, Medical Faculty and University Hospital Düsseldorf, Heinrich-Heine-University Düsseldorf, Düsseldorf, Germany; 5 Department of Neurology with Heimer Institute for Muscle Research, University Hospital Bergmannsheil, Bochum, Germany; 6 Department of Neurology, Center for Translational Neuro- and Behavioral Sciences (C-TNBS), University Hospital Essen, University of Duisburg-Essen, Essen, Germany; 7 Institute of Myology, Neuromuscular Morphology Unit & Neuromuscular Diseases Reference Center Nord/Est/Ile-de-France, Sorbonne Université, INSERM, GHU Pitié-Salpêtrière, Paris, France; 8 Department of Neuropathology, Sorbonne University, APHP, Pitié-Salpêtrière Hospital, 75013 Paris, France; 9 Children’s Hospital of Eastern Ontario Research Institute, University of Ottawa, Ottawa, ON Canada

**Keywords:** Myositis, Anti-CD30, Brentuximab vedotin, Chemotherapy, Hodgkin lymphoma

## Abstract

**Objective:**

Immune-related adverse events (irAEs) are recognized in oncology, particularly with immune checkpoint inhibitors and other targeted therapies. Brentuximab Vedotin (BV), is an anti-CD30 antibody–drug conjugate- its association with immune-mediated myositis remains unexplored. We report a case of an adolescent with Hodgkin lymphoma (HL) who developed neuropathy and myositis following BV therapy.

**Materials & methods:**

The diagnostic work-up included MRI as well as microscopic analyses (histology, electron microscopy, and immunostainings including CD30 and MxA) of a gastrocnemius muscle biopsy. Proteomic analysis was also performed on the same biopsy, and paradigmatic protein dysregulations were validated through immunostaining. Serum NCAM1 levels were measured using ELISA.

**Results:**

The patient, diagnosed with HL at 15 years, developed neuropathy after Vincristine treatment and was switched to BV. During BV therapy, she experienced progressive muscle weakness and foot drop, leading to discontinuation. MRI confirmed myositis, and biopsy revealed neurogenic and inflammatory changes with complement deposition and mitochondrial dysfunction. Proteomics showed upregulation of inflammatory relevant proteins, with HPRT1 (749.43-fold) being the most increased one. Intravenous immunoglobulin (IVIG) therapy improved muscle strength.

**Discussion:**

Myositis following BV therapy has not been reported. Findings suggest an immune-mediated mechanism with B-cell involvement. Given the response to IVIG, B-cell-directed therapies may be beneficial. This case identifies BV-induced myositis as a novel irAE.

**Supplementary Information:**

The online version contains supplementary material available at 10.1186/s40478-025-02056-2.

## Introduction

Therapeutic strategies for oncological diseases have occasionally been linked to the development of immune-related adverse events (irAEs). A striking example is the manifestation of muscle inflammation (myositis) in patients undergoing targeted therapies especially with checkpoint inhibitors [9, 14]. Brentuximab is not a checkpoint inhibitor, but an antibody–drug conjugate (ADC) used to treat cancers like Hodgkin lymphoma (HL) and anaplastic large cell lymphoma. It combines an anti-CD30 monoclonal antibody with a chemotherapy drug, targeting CD30 on cancer cells and delivering the chemotherapy directly to them, resulting in cell destruction. In contrast, checkpoint inhibitors (e.g., Pembrolizumab & Nivolumab) act by blocking immune checkpoint proteins like PD-1 or PD-L1, which normally prevent the immune system from attacking cancer cells. By inhibiting these checkpoints, the immune system is "released" to better recognize and destroy cancer cells. Brentuximab targets cancer cells directly, while checkpoint inhibitors manipulate immune responses.

We report on a female late adolescent oncological patient, who underwent treatment with Vincristine, subsequently receiving BV for HL and developed neuropathy followed by myositis with substantial complement deposition and mitochondrial vulnerability. Myositis was treated with corticosteroids (pulse therapy) followed by immunoglobulins (IVIG).

## Methods

Routine diagnostic work-up included muscle MRI and histological studies on 7 µm cyrosections of gastrocnemius muscle in addition to electron microscopic studies (EM) on the muscle biopsy. To obtain a profound understanding of immunopathologic patterns and organelle vulnerabilities in the muscle biopsy of our patient, we next performed unbiased proteomic profiling using a data-independent-acquisition approach as described previously [11]. Paradigmatic findings were confirmed by immunofluorescence (Suppl. Tab. 1). To address presence of active neuropathy during clinical manifestation of myositis, ELISA-based studies were carried out on serum for NCAM1 according to the respective manufacturer´s instructions (R&D Systems; DY2408). Applied Myositis antibody panel (commercial laboratory) included: anti-Ku, -Jo-1, -PL-7, -PL-12, -EJ, -SRP, -TIF1 gamma, -MDA5, -Mi2 alpha, -Mi-2 beta, -NXP2, -OJ, -PM-Scl100, -PM-Scl75, -SAE1, and -Ro-52.

Analytical procedures and genetic studies were undertaken after obtaining written consent of the patient. Experimental studies were approved by the local ethics committee (19–9011-B0).

## Results

**Fig. 1:**
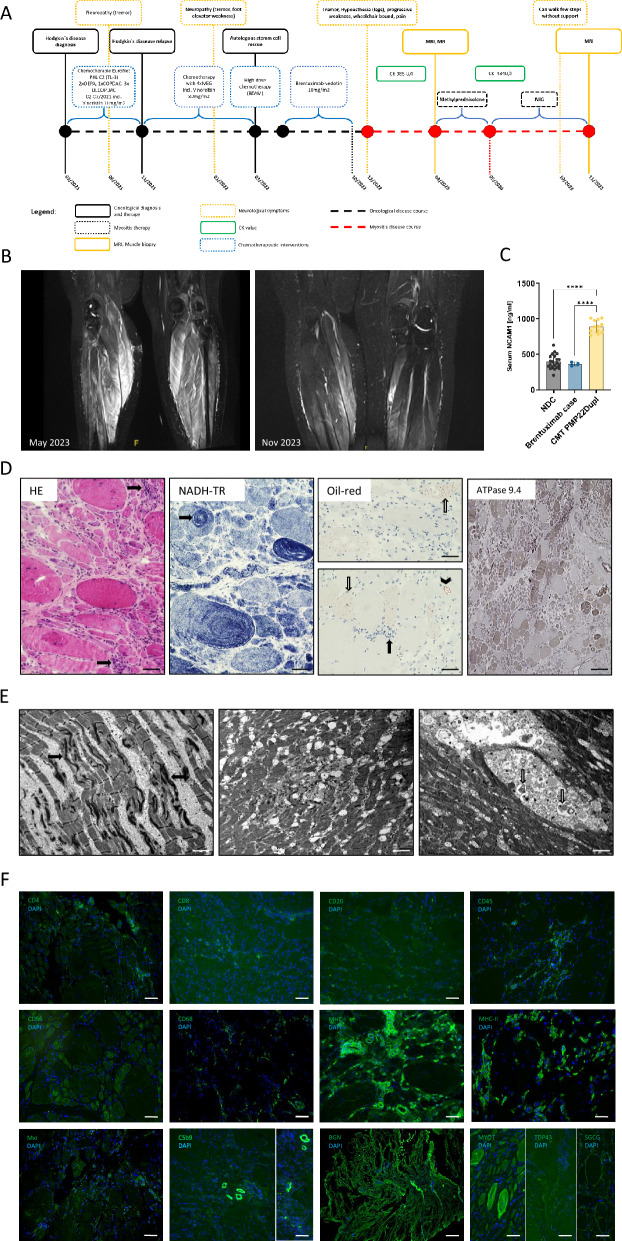
Clinical findings in the index patient: **A** Timeline of preceding events, in-hospital stay and follow-up. **B** MRI of the lower thigh demonstrating myositis of the gastrocnemius muscle on both sides under BV treatment (left) and 6 months after termination of therapy (right). **C** Results of ELISA-based NCAM1 quantification in sera derived from healthy donors (left), our patient (middle; measure in technical triplicates) and Charcot-Marie-Tooth type 1A (CMT1A; *PMP22* duplication) cases (right) included as disease controls. **D** Histological stains on gastrocnemius biopsy revealing neurogenic changes including numerous atrophic, partially angular shaped and grouped fibres (H&E and ATPase 9.4 stains) and myogenic-inflammatory changes (black arrows in H&E and Oil red stains) occasionally accompanied by lipid droplet accumulation within the sarcoplasm (white arrows in Oil red stains) or vacuoles (arrowhead in Oil red stains) as well as ringed fibres (black arrow in NADH-TR stain). Scale bars for H&E, NADH-TR & Oil red = 80 µm and for ATPase 9.4 stain = 200 µm. **E** Ultra-structural studies on same biopsy showing sarcomeric disintegrations (black arrows), altered mitochondrial morphology, increase of lipid droplets and build-up of protein aggregates within vacuoles (white arrows). Scale bars = 2 µm. **F** Immunofluorescence studies on the same biopsy for inflammation and myofibrosis markers. Sparse CD4, CD8, CD20, CD45, and CD68 positive cells are seen in addition to increase of MHC-II and MxA in the vicinity of damaged muscle fibres. Small regenerating fibres also express CD56. Immunostaining for MHC-I and C5b-9 displaying complement deposition on scattered muscle fibres and capillaries. Scale bars = 100 µm. Immunostaining for biglycan (BGN) showing considerable increase within the extracellular matrix. Scale bar = 200 µm. Further routine stains showed irregular Myotilin (MYOT) distribution in degenerating fibres, punctuate TDP43-immunoreactivity in the perimysium and within few muscle fibres as well as irregular sarcoplasmic deposition of gamma-Sarcoglycan (SCGC) in some muscle fibres. Scale bars = 100 µm

The patient was diagnosed with HL at the age of 15 and subsequently began a standardized chemotherapy regimen according to the EuroNet PHL C2 (TL-3) protocol, which included Vincristine. Because of clinical signs of neuropathy (hand-tremor) and severely reduced CYP3A5-activity (homozygous *3/*3 genotype), Vincristine-treatment was terminated. As she was diagnosed with HL-relapse 10 months after the initial diagnosis, relapse chemotherapy (4 × IGE (Ifosfamide, gemcitabine) & Vinorelbin 80 mg/m2) followed by autologous stem cell transplantation (ASCT) in combination with high dose-chemotherapy protocol (BEAM) was performed. Three months after, antibody-based therapy (anti-CD30) with BV as consolidation treatment for relapses of HL after ASCT was initiated (Fig. 1A). During this therapy, the patient complained of numbness in her feet and walking problems due to bilateral foot drop, which lead to early termination of BV-therapy (22 weeks after initiation). In addition to sensorimotor neuropathy, signs of distal and proximal muscular weakness occurred over the period of two months after termination of the BV-therapy: she reported decline in her muscular strength, calve-pain and inability to climb stairs or to lift from the sitting position. Strength of upper extremities remained normal. No skin lesions were present. Laboratory findings revealed elevated creatinine kinase (CK) levels of 985 U/l (normal range: 34–147 U/l). Whole-body MRI demonstrated signs of myositis in both gastrocnemius muscles (Fig. 1B). NCAM1 as a marker for active neuropathy (although not used in routine clinical practice but for research purposes) [6] was unchanged in serum of our patient (Fig. 1C). Serum testing revealed negative results for myositis specific or associated antibodies, thus a gastrocnemius muscle biopsy was collected for further diagnostic work-up. The biopsy demonstrated pathological skeletal muscles with neurogenic (numerous atrophic, partially angular shaped and grouped fibres) and myogenic-inflammatory changes occasionally accompanied by lipid droplet accumulation and ringed fibres (Fig. 1D). Electron microscopy unveiled altered sarcomeric and mitochondrial morphology, increase of lipid droplets and build-up of protein aggregates within vacuoles (Fig. 1E). Immunohistochemistry showed CD4, CD8, CD20, CD45, and CD68 cell infiltration as well as MHC-I, MCH-II, MxA and biglycan (BGN) overexpression and terminal complement (C5b-9) deposition on capillaries (Fig. 1F). To address whether muscle inflammation is associated with HL, we moreover performed CD30 staining studies also utilizing muscle biopsy specimens derived from juvenile dermatomyositis (jDM, n = 2), immune-mediated necrotizing myositis (IMNM, n = 1) and Duchenne muscular dystrophy (DMD, n = 1) patients serving as disease controls—all of them non-paraneoplastic. A muscle biopsy derived from a DMD—patient was included based on the well-known presence of inflammation in the molecular aetiology of the myopathology. Of note, increase of CD30 cells was also observed on these biopsies serving as diseases controls (supplementary Fig. 1).

Based on these findings, therapy with pulsed intravenous methylprednisolone was started. As clinical condition remained unchanged, therapy was switched to intravenous immunoglobulins (IVIG) (2 g/kg), applied once monthly and her muscular strength and function improved three months afterwards. The patient was able to walk a few steps without support and without pain. Follow-up MRI (whole-body) 6 months after initiation of the therapy showed reduction in T2 weighted STIR signal of the previously affected muscles (Fig. 1B). No signs of HL-relapse were present (PET2 negative). At last clinical follow up, 17 months after the onset of her muscular weakness, the patient exhibited full and unrestricted mobility. She demonstrated mildly diminished distal muscle strength in her feet, accompanied by absent Achilles tendon reflexes and decreased patellar reflexes. Nerve conduction velocities (NCV) showed neurogenic pattern in her lower extremities and normal NCV in her upper extremities. Electromyogram showed no spontaneous activity.

**Fig. 2:**
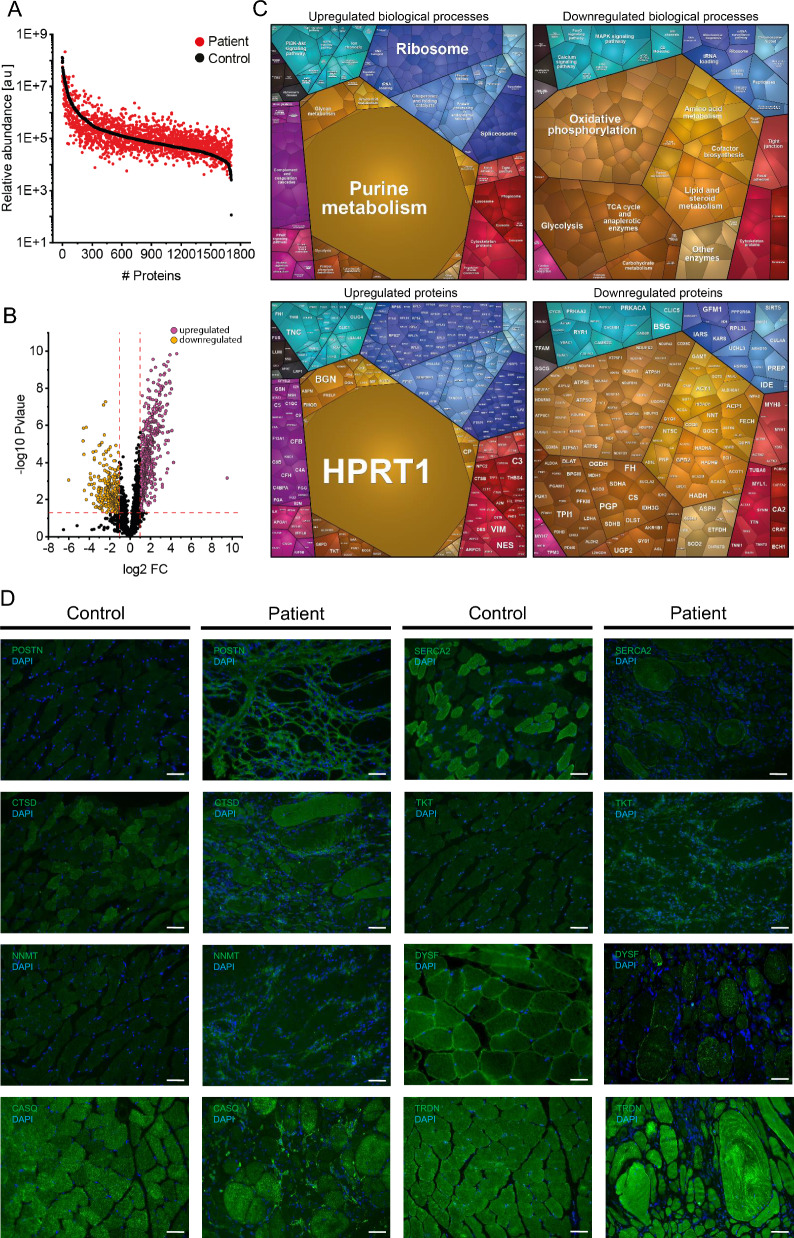
Results of proteomic profiling and validation studies on gastrocnemius muscle of the index patient: **A** Abundance plot showing the dynamic range of all proteins identified in proteins extracts of gastrocnemius muscle via liquid chromatography coupled to tandem mass spectrometry based on their relative quantification using always the 3 highest abundant peptides for each protein, allowing protein comparison within an experiment. All identified proteins of the controls (black) are sorted with decreasing abundance while the patient (red) was plotted in the same order to directly compare the different abundances. All identified proteins cover a dynamic range of eight orders of magnitude. **B** Volcano plot highlighting statistically significant increased proteins (purple dots) as well as decreased proteins (yellow dots). (**C**) Proteomaps-based in silico analyses of dysregulated proteins for upregulated (left panel) and downregulated (right panel) biological processes and related proteins separately. (**D**) Immunofluorescence studies of paradigmatic proteins (Periostin = POSTN, Cathepsin D = CTSD, Nicotinamide N-methyltransferase = NNMT, Sarcoplasmic/endoplasmic reticulum calcium ATPase 2 = SERCA2, Transketolase = TKT & Dysferlin = DYSF, Calsequestrin = CASQ, Triadin = TRDN) to validate proteomic findings making use of another analytical approach. Scale bars = 80 µm

Proteomic profiling of the muscle biopsy was conducted to elucidate the precise molecular signature underlying the muscle pathology, revealing a statistically significant increase of 489 proteins and a decrease of 301 proteins (Suppl. Tab. 2). The most markedly upregulated protein was HPRT1 (749.43-fold) (Fig. 2C). The increased proteins moreover include various factors linked to the activation of the complement system (Fig. 2C). Proteomaps-based pathway analysis revealed that the upregulated proteins additionally influence various cellular processes, including signalling pathways and protein synthesis, folding and degradation. Decreased proteins indicate a profound vulnerability of mitochondrial proteins involved in oxidative phosphorylation in addition to such involved in amino acid, lipid and steroid metabolism as well as glycolysis among others (Fig. 2C). Immunofluorescence studies of paradigmatic proteins covering different molecular functions (DYSF, POSTN, CATD, TKT, NNMT & SERCA2) were performed and showed similar dysregulations as proteomics (Fig. 2D).

## Discussion and conclusions

Although immune-related adverse events (irAEs) are recognized in oncological treatments, myositis as a consequence of anti-CD30 therapy has not been reported yet. Our patient was treated with Vincristine, followed by BV. Vincristine, commonly used in childhood malignancies, primarily causes peripheral neuropathy (PN) as irAE. Its metabolism depends on CYP3A4 and CYP3A5 enzymes, but recent studies suggest neurotoxicity is dose-independent and not solely determined by genotype [7]. However, genetic testing in our patient revealed a homozygous *3/*3 genotype, predisposing her to neuropathy, leading to therapy termination and transition to BV. During subsequent BV treatment, PN symptoms worsened, accompanied by muscular pain. Notably, 20% of pediatric high-risk Hodgkin lymphoma (HL) patients treated with BV develop PN [3]. Unlike a typical BV or Vincristine induced neuropathy, the patient exhibited both proximal and distal muscle weakness in the lower extremities. MRI and muscle biopsy revealed signs of myositis in her gastrocnemius muscles—a condition not previously linked to BV therapy. Elevated NCAM1 expression may signify denervation-induced muscle regeneration, serving as a potential biomarker for monitoring disease progression or therapeutic response [6]. However, serum studies showed no NCAM1 changes in our patient, suggesting predominant muscular involvement instead of active neuropathy. Histological analysis demonstrated an inflammatory myopathology with fibrotic remodeling, confirmed by biglycan and periostin staining.

In some hereditary myopathies, such as *ANO5* or *CAV3*, increased T2-weighted MRI hyperintensity in gastrocnemius muscle as well histopathological evidence of muscle inflammation may be present [5, 12]. Consequently, genetic analysis for hereditary myopathies was conducted in our patient and revealed no pathological findings in turn hinting toward the presence an acquired muscle pathology.

Myositis is a known irAE of immune checkpoint inhibitors, typically involving T-cell and macrophage infiltration, complement deposition, and MHC-I overexpression [14]. While BV is not a checkpoint inhibitor, similar myopathologies were observed in our patient, with additional B-cell (CD20 & CD45) infiltration. Important to note, although CD30 is a surface molecule recognized by Ki-1 monoclonal antibody on Hodgkin and Sternberg-Reed cells in patients with HL, CD-30 can also be expressed in further cells, including T cells, B cells and activated human CD4 + T-cell clones. This accords with our finding of CD30 cells in muscle biopsies across different acquired and one genetic condition (jDM, IMNM, DMD). Thus, these combined staining studies may indicate a general role of CD30 expressing cells in the context of muscle inflammation rather than indicating invasion of tumour cells. Along this line, a previous study highlighted that in blood of DM/polymyositis (PM) patients, CD30 levels were significantly elevated compared to normal controls [16].

Proteomic profiling further indicated inflammatory pathology with complement activation and a significant HPRT1 increase (749.43-fold). HPRT1 enhances chemoresistance via the MMP1/PI3K/AKT pathway [15] and plays a role in tumorigenesis and purine metabolism in Epidermal Growth Factor Receptor (EGFR)-mutant lung adenocarcinoma [4]. This increase may thus correlate with chemotherapy resistance. Additionally, HPRT1 deficiency is linked to mitochondrial dysfunction [4], which aligns with observed mitochondrial abnormalities in our patient’s muscle biopsy, including ringed fibres and lipid droplet accumulation. Mitochondrial dysfunction is a known driver of myositis [2]. Also, other dysregulated proteins, such as NNMT (increased), are associated with mitochondrial dysfunction, fibrosis, and cancer metabolism [10, 17].

BV-induced PN improves in up to 72% of patients over time [1]. In our case, BV was discontinued to prevent progression. The patient remains in remission for over 30 months after treatment termination. Some patients benefit from immunotherapy, including corticosteroids, plasma exchange, or IVIG, reinforcing the immune-mediated nature of PN [13].

Our patient responded well to IVIG, further supporting the concept of an inflammatory pathology. Given the presence of B-cell infiltration in the biopsy, B-cell-directed therapy (e.g. Rituximab) could be considered. Unlike adults, myositis as a paraneoplastic syndrome is rare in pediatric malignancies [8]. Follow-up MRI (whole-body, PET-MRI) six months after initiation of the therapy showed reduction in T2 weighted STIR signal of the previously affected muscles (Fig. 1B). No signs of HL-relapse were present (PET2 negative).

This case highlights the first reported instance of myositis in addition to neuropathy as an immune-related adverse event of BV, suggesting an inflammatory pathology with B-cell involvement and potential implications for targeted immunotherapy.

## Supplementary Information

Supplementary Material 1 Immunofluorescence studies of CD30 in quadriceps muscle biopsy specimens derived from our patient, two juvenile dermatomyositis (jDM), one immune-mediated necrotizing myopathy (IMNM) and one Duchenne muscular dystrophy (DMD) patient, respectively. In both, CD30 show increase in the vicinity of damaged muscle fibres. Scale bars = 40 µm).

Supplementary Material 2 (List of primary antibodies used in our study)

Supplementary Material 3 (Overview of proteomic findings on gastrocnemius muscle of the patient).

## Data Availability

Data is provided within the manuscript or supplementary information files.

## Abbreviations

ADC: Antibody–drug conjugate
ASCT: Autologous stem cell transplantation
BEAM: Carmustine, etoposide, cytarabine, melphalan
BGN: Biglycan
BV: Brentuximab vedotin
CATD: Cathepsin D
CD30/TNFRSF8: Tumor necrosis factor receptor superfamily member 8
CK: Creatinine kinase
CMT1A: Charcot-Marie-Tooth 1A
DMD: Duchenne muscular dystrophy
CYP: Cytochrome
DYSF: Dysferlin
EGFR: Epidermal growth factor receptor
EJ: Glycyl-tRNA synthetase
ELISA: Enzyme-linked immunosorbent assay
EM: Electron microscopic studies
HL: Hodgkin lymphoma
HPRT1: Hypoxanthine phosphoribosyltransferase 1
IGE: Ifosfamide, gemcitabine
IMNM: Immune-mediated necrotizing myopathy
irAEs: Immune-related adverse events
IVIG: Intravenous immunoglobulin
jDM: Juvenile dermatomyositis
Jo-1: Histidyl transfer ribonucleic acid (tRNA) synthetase
MDA5: Anti-melanoma differentiation-associated gene 5
MHC: Major histocompatibility complex
MMP1: Matrix metalloproteinases
MRI: Magnetic resonance imaging
MxA/Mx1: Myxovirus resistance protein A/Interferon-induced GTP-binding protein Mx1
NCAM1: Neural cell adhesion molecule 1
NNMT: Nicotinamide *N*-methyltransferase
NCV: Nerve conduction velocities
NXP 2: Nuclear matrix protein 2
OJ: Isoleucyl-tRNA synthetase
PD: Programmed cell death protein
PD-L: Programmed cell death ligand
PET2: Positron emission tomography scan after 2 cycles of chemotherapy
Pl3K: Phosphatidylinositol 3-kinase
PL-7: Threonyl-tRNA synthetase
PL-12: Anti-alanyl-tRNA synthetase
PM: Polymyositis
PN: Peripheral neuropathy
POSTN: Periostin
SAE1: Small ubiquitin-like modifier-1 activating enzyme
SERCA2: Sarcoplasmic/endoplasmic reticulum Ca^2^^+^-ATPase
SRP: Signal recognition particle
STIR: Short-tau-inversion-recovery-sequenz
TIF1: Transcription intermediary factor 1
TKT: Transketolase
