# Supplementary figures and images for: Co-occurrence of myositis and neuropathy after anti-CD30 therapy in a late-adolescent Hodgkin lymphoma patient
Source: Acta Neuropathol Commun. 2025 Jun 28;13:140. doi: 10.1186/s40478-025-02056-2 (PMC12205510; doi:10.1186/s40478-025-02056-2)

Control

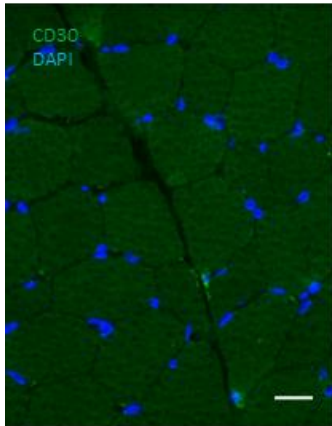

Our patient

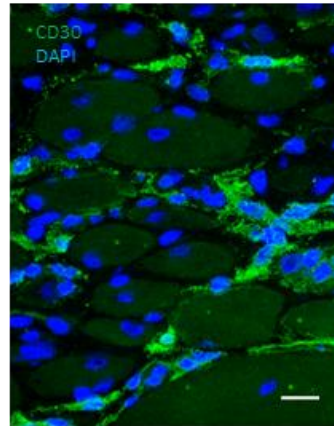

jDM

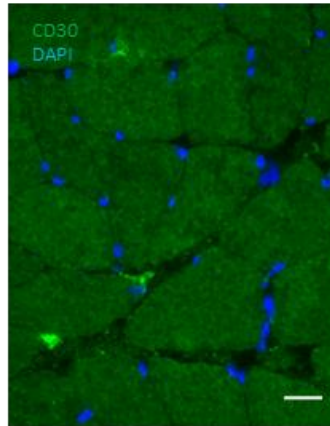

IMNM

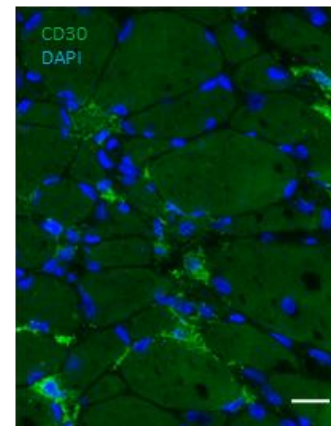

DMD

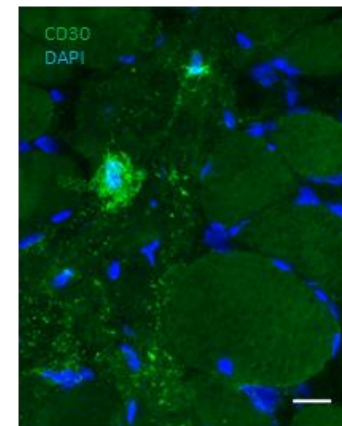

Supplement: Supplementary file 1 — Supplementary Material 1 Immunofluorescence studies of CD30 in quadriceps muscle biopsy specimens derived from our patient, two juvenile dermatomyositis (jDM), one immune-mediated necrotizing myopathy (IMNM) and one Duchenne muscular dystrophy (DMD) patient, respectively. In both, CD30 show increase in the vicinity of damaged muscle fibres. Scale bars = 40 µm). [file 40478_2025_2056_MOESM1_ESM.pdf]
