# Supplementary material for: Co-occurrence of myositis and neuropathy after anti-CD30 therapy in a late-adolescent Hodgkin lymphoma patient
Source: Acta Neuropathol Commun. 2025 Jun 28;13:140. doi: 10.1186/s40478-025-02056-2 (PMC12205510; doi:10.1186/s40478-025-02056-2)
Supplement: Supplementary file 2 — Supplementary Material 2 (List of primary antibodies used in our study) [file 40478_2025_2056_MOESM2_ESM.pdf]

Primary antibody	Supplier	Dilution
anti-CD4 (mouse)	Dako M0716	1:50
anti-CD8 (mouse)	Leica NCL-L-CD8-4B11	1:20
anti-CD20 (mouse)	Leica	1:200
anti-CD45 (mouse)	Dako M0701	1:50
anti-CD56/ NCAM1 (mouse)	Abcam ab9018	1:100
anti-CD68 (mouse)	Dako M0718	1:100
anti-MHCI (mouse)	Dako M0736	1:100
anti-C5b9 (mouse)	Dako M0777	1:25
anti-Biglycan (rabbit)	Thermo Scientific PA5-76821	1:100
anti-Myotilin (mouse)	Leica NCL-MYOTILIN	1:50
anti-TDP43 (mouse)	Abnova H00023435-M01	1:100
anti-gamma-Sarcoglycan (mouse)	DAG35 gift of Kevin Campbell	1:25
anti-Periostin (rabbit)	Abcam ab14041	1:100
anti-Cathepsin D (mouse)	Abcam ab6313	1:100
anti- Nicotinamide N-methyltransferase (mouse)	Santa Cruz sc-376048	1:100
anti-Calsequestrin (rabbit)	Abcam ab185220	1:100
anti- Sarcoplasmic/endoplasmic reticulum calcium ATPase 2 (mouse)	Sigma S1439	1:100
anti-Transketolase (mouse)	Santa Cruz SC-390179	1:100
anti-Dysferlin (mouse)	Leica NCL-HAMLET	1:5
anti-Triadin (rabbit)	Abcam ab131631	1:100
